# Supplementary material for: Microstimulation of human somatosensory cortex evokes task-dependent, spatially patterned responses in motor cortex
Source: Nat Commun. 2023 Nov 10;14:7270. doi: 10.1038/s41467-023-43140-2 (PMC10638421; doi:10.1038/s41467-023-43140-2)
Supplement: Supplementary file 1 — Supplementary Information [file 41467_2023_43140_MOESM1_ESM.pdf]

## SUPPLEMENTARY FIGURES

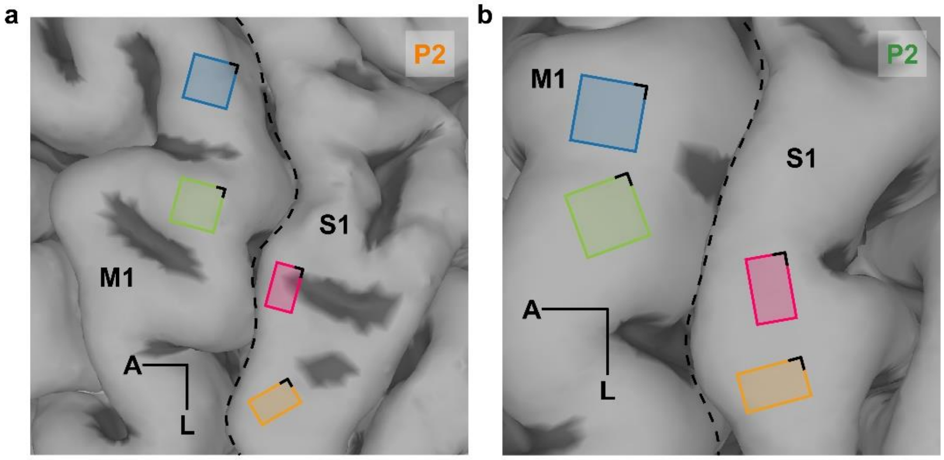


*Supplementary Figure 1. Implant locations for P2 (A) and P3 (B). See Figure 1 for C1.*

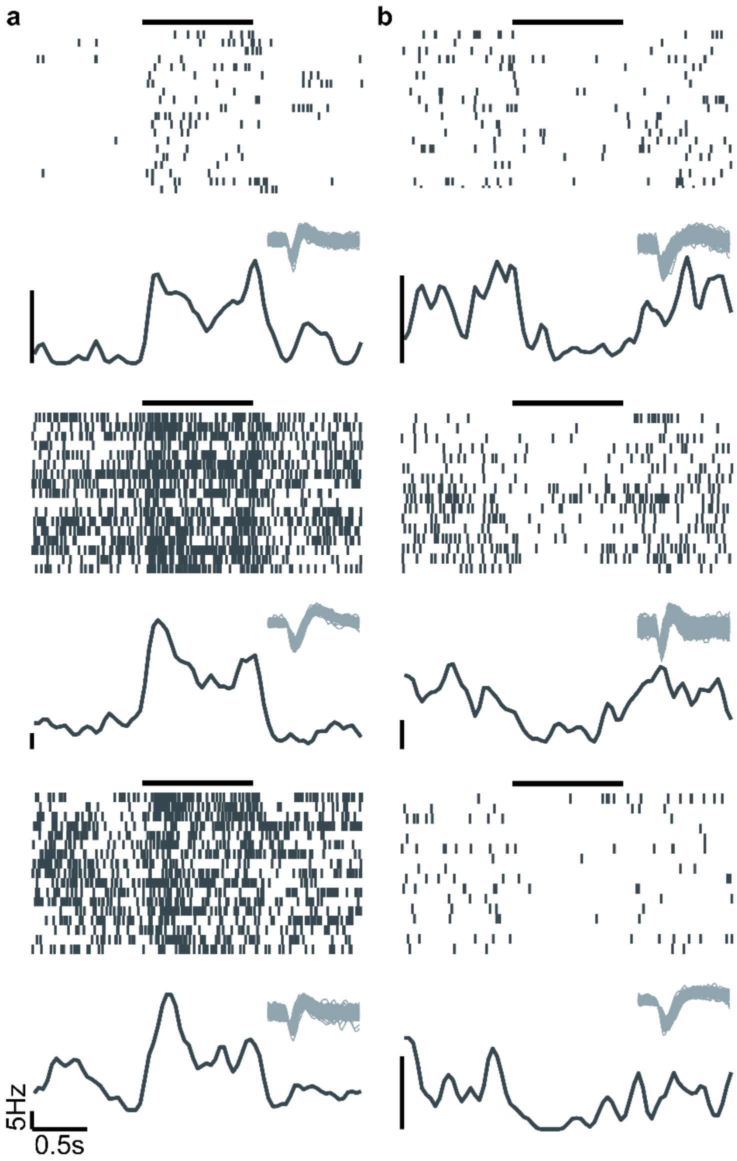


*Supplementary Figure 2. Single unit responses in M1 during ICMS delivered to S1. A) Responses of three example neurons (inset: sorted waveforms) that were excited by stimulation (from top to bottom: C1, P3, P3). B) Responses of three example neurons that were inhibited by stimulation (C1, P2, P3).*

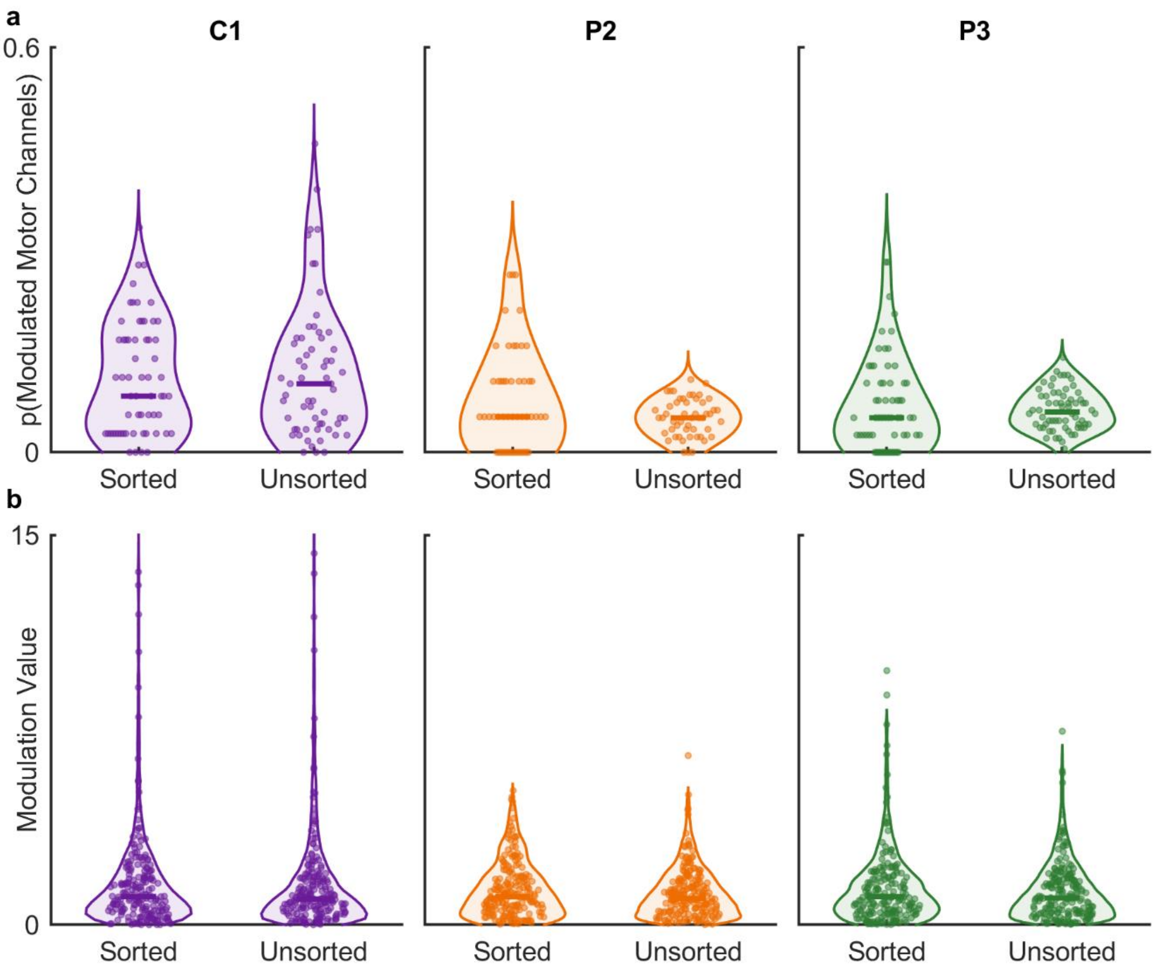


*Supplementary Figure 3. Prevalence of ICMS-evoked activity in motor cortex is similar for sorted and unsorted units. A| Proportion of motor channels significantly modulated by stimulation channels (each dot represents a stimulation channel) for sorted and unsorted units (N = 36, 19, and 39 sorted units for participants C1, P2, and P3, respectively). B| Distribution of absolute modulation values for all pairs of motor and stimulation channels. Unlike in Figure 2B, where modulation values are averaged for each motor channel, the modulation value for each motor-sensory pair is shown separately given the small number of sorted units.*

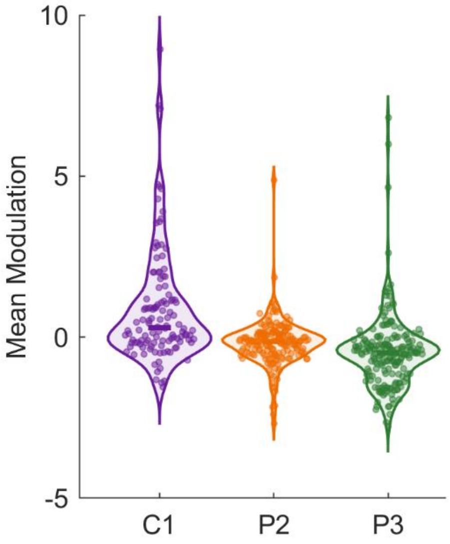


*Supplementary Figure 4. Mean ICMS-driven modulation for each motor channel. This figure shows the raw (signed) modulation values rather than the absolute ones.*

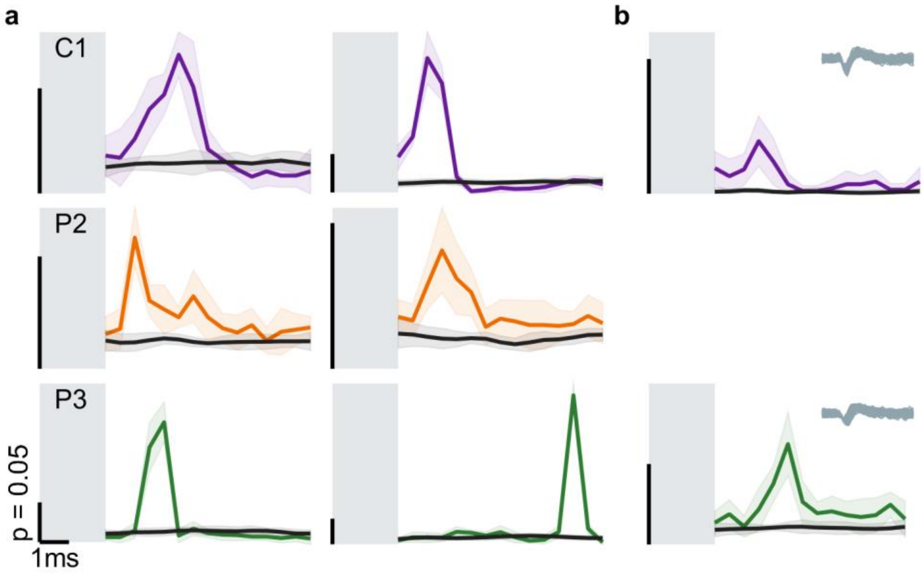


*Supplementary Figure* 5*. ICMS to S1 evokes short-latency, pulse-locked responses in M1. A| Pulse triggered average (PTA) of the M1 responses evoked ICMS to S1 (colored). As a control, we computed a sham pulse triggered average (at the same pulse frequency) during baseline (black). Temporally precise responses occur at varying latencies across motor channels and participants. Each row shows two example PTAs from different S1-M1 electrode pairs for each participant (C1, P2, P3). The probability of a spike occurring in each 0.5 ms bin is shown on the y-axis Error bars represent bootstrapped standard error. B| Example PTAs for sorted units from participants C1 and P3. No sorted units with phase-locking were observed in P2.*

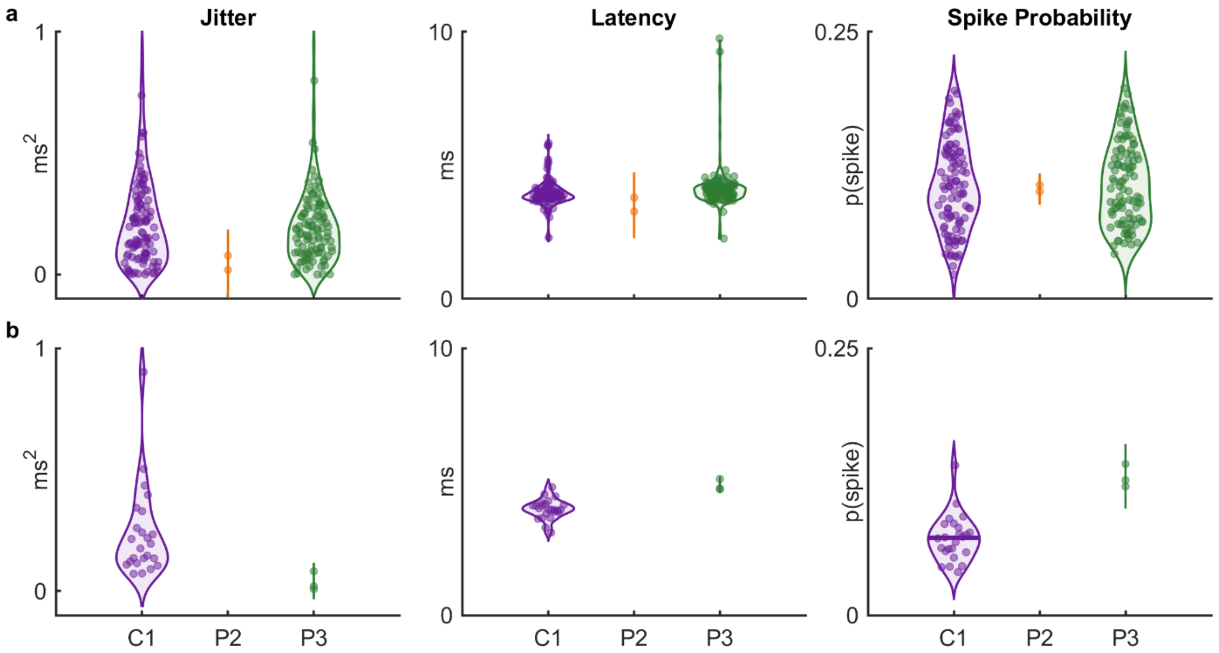


*Supplementary Figure 6. Characteristics of pulse-locked responses. A| Characteristics of pulse-locked responses of unsorted units for each participant. Each dot represents a motor channel-stimulation channel pair. The three metrics – jitter, latency, and spiking probability (the proportion of times a pulse evoked a spike within a 1-ms window centered at the time of highest spiking probability) – were distributed unimodally, precluding classification of pulse-locked responses as reflecting antidromic or orthodromic activation. If spikes with jitter less than 0.1 ms$^2$ are considered to reflect antidromic activation (cf. refs. [37,38]), these responses reflect both antidromic and orthodromic activity, with a far greater prevalence of orthodromic activity. B| The same metrics in sorted units are consistent with responses of unsorted units.*

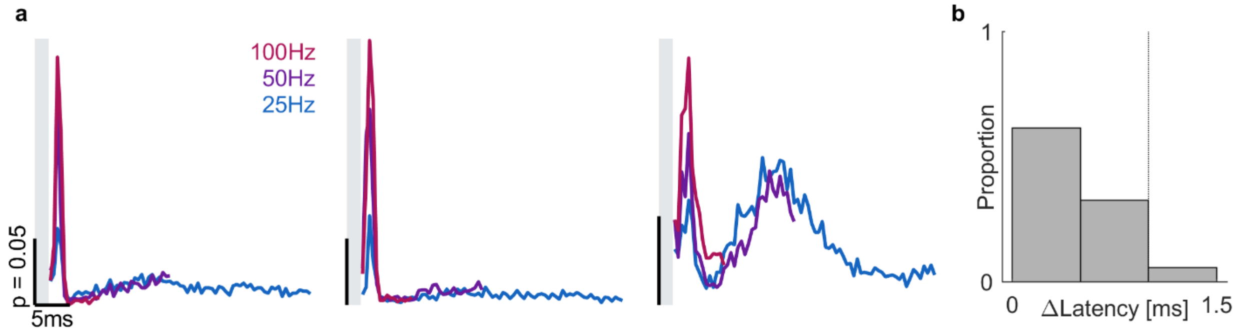


*Supplementary Figure 7. Pulse-triggered average (PTA) of the responses evoked by ICMS at three frequencies in C1. A| Three example motor channels show the preserved initial response latency regardless of stimulation frequency. Some channels demonstrate a secondary, longer latency but lower probability response that is obscured during high frequency stimulation. B| Distribution of the differences in peak latency times across the 3 frequencies. Vertical line indicates the temporal resolution of the analysis. All latency differences fall within the temporal resolution of the analysis.*

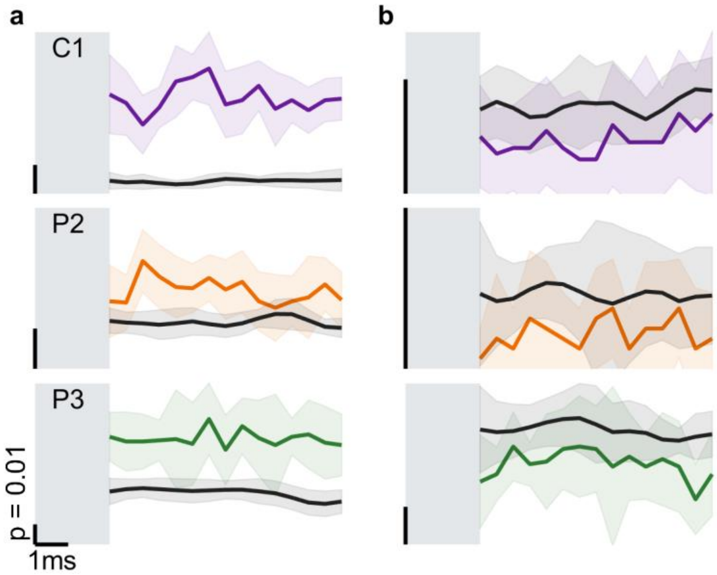


*Supplementary Figure 8. Pulse triggered average (PTA) during ICMS (colored, mean +/-bootstrapped standard error) from channels that were modulated by stimulation but did not exhibit pulse locking in their response. As a control, we computed a sham pulse triggered average (at the same pulse frequency) during baseline (black). The gray area indicates time during which recording was blanked to eliminate the stimulation artifact. The y-axis denotes the probability of a spike occurring in each bin. A| Channels that were significantly excited by stimulation. B| Channels that were significantly inhibited by stimulation. Each row shows example PTAs for each participant (C1, P2, P3).*

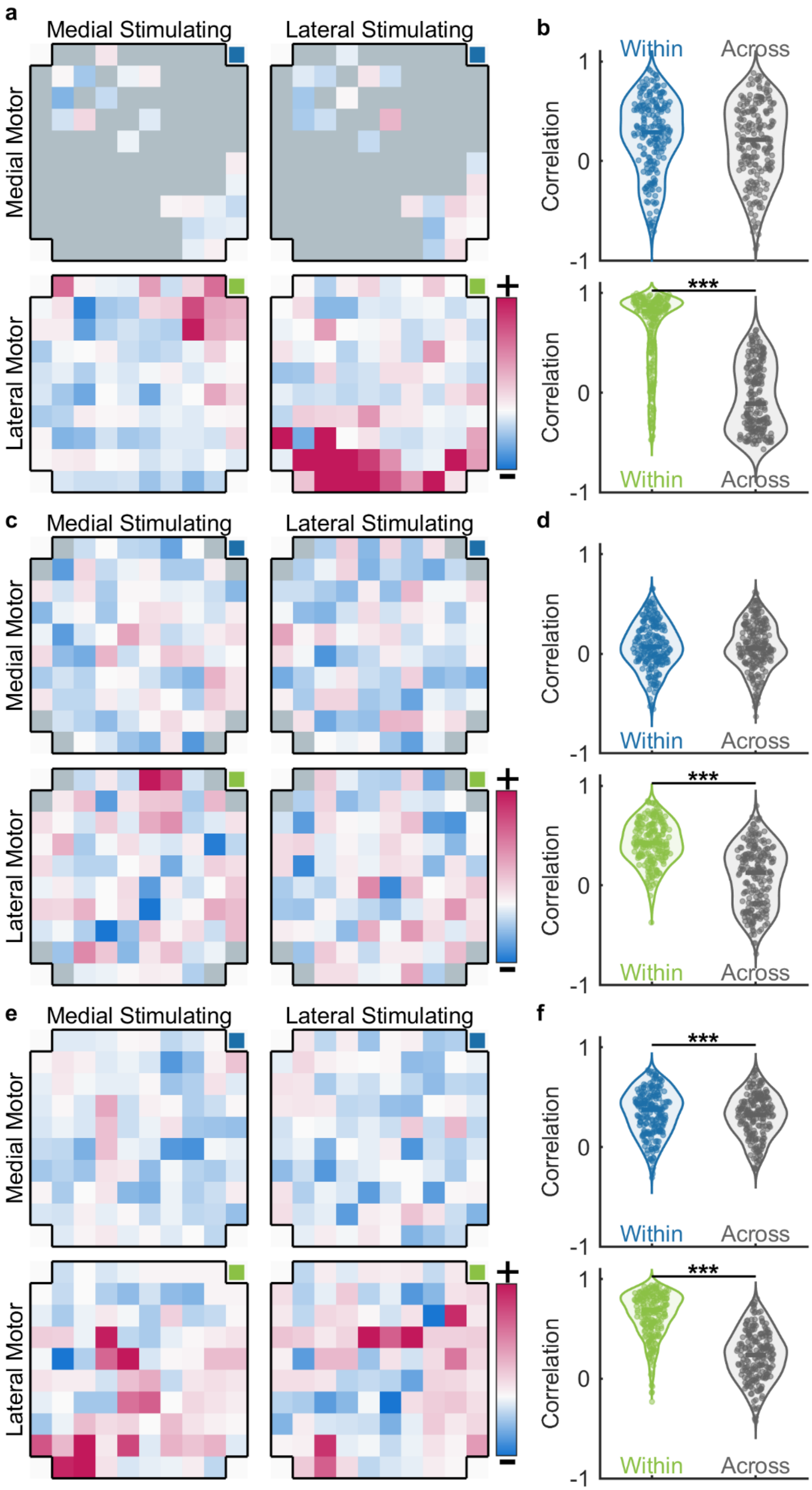

a
Medial Stimulating
Lateral Stimulating
Medial Motor
Lateral Motor
+
-
b
Within
Across
Correlation
1
0
-1
***
c
Medial Stimulating
Lateral Stimulating
Medial Motor
Lateral Motor
d
Within
Across
Correlation
e
Medial Stimulating
Lateral Stimulating
Medial Motor
Lateral Motor
f
Within
Across
Correlation

*Supplementary Figure 9. Spatial patterning of ICMS-evoked M1 activation. A| Stimulation through an example channel on the medial somatosensory array and lateral sensory array for C1. Adjacent channels are separated by 400 µm. Blue and green squares in upper right of arrays indicate their orientation on the brain (Figure 1A). Grey squares denote inactive motor channels. The color bars represent the z-scored stimulation-related modulation scale (-5 to 10). B| Correlation between the spatial pattern of activation evoked in M1 by pairs of stimulation channels belonging to the same stimulation array (within array) or different arrays (across array) for C1. Asterisks indicate significance ($p < 0.001$ rank-sum test). C| Same as A for P2. D| Same as B for P2. E| Same as A and C for P3. F| Same as B and D for P3. The lateral motor arrays for all three participants show significantly higher correlation between pairs of stimulation electrodes on the same sensory array than on different sensory arrays, but this patterning is much stronger in C1 and P3 than in P2.*

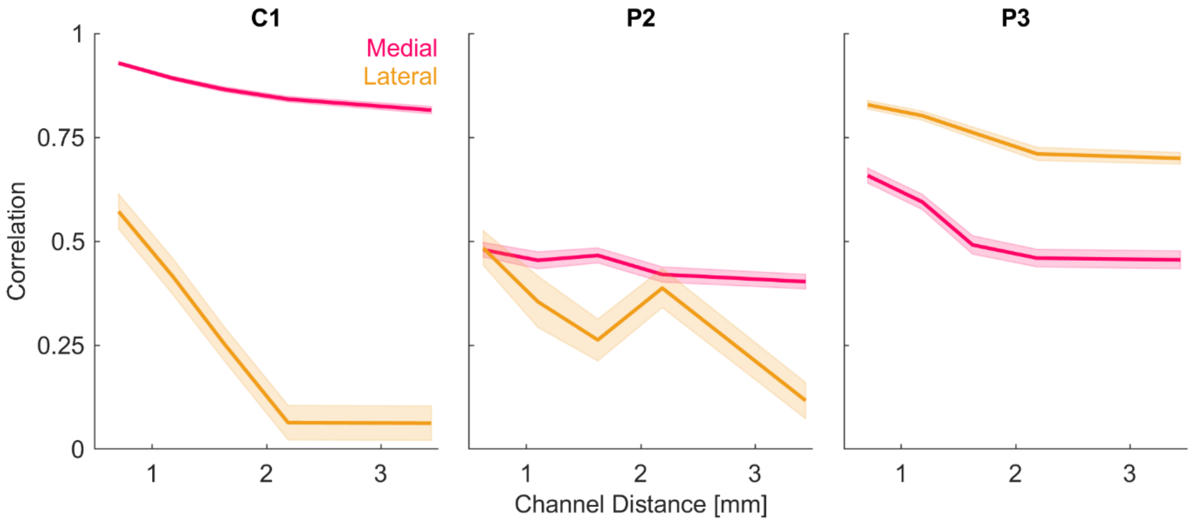


*Supplementary Figure 10. Correlation between the spatial pattern evoked in the lateral motor array as a function of distance between two stimulating electrodes in the medial (pink) and lateral (orange) sensory arrays (mean +/- SEM). The spatial pattern of activation evoked by two electrodes tends to be more similar when the two electrodes are nearby. Correlations were lower for participant P2 overall.*

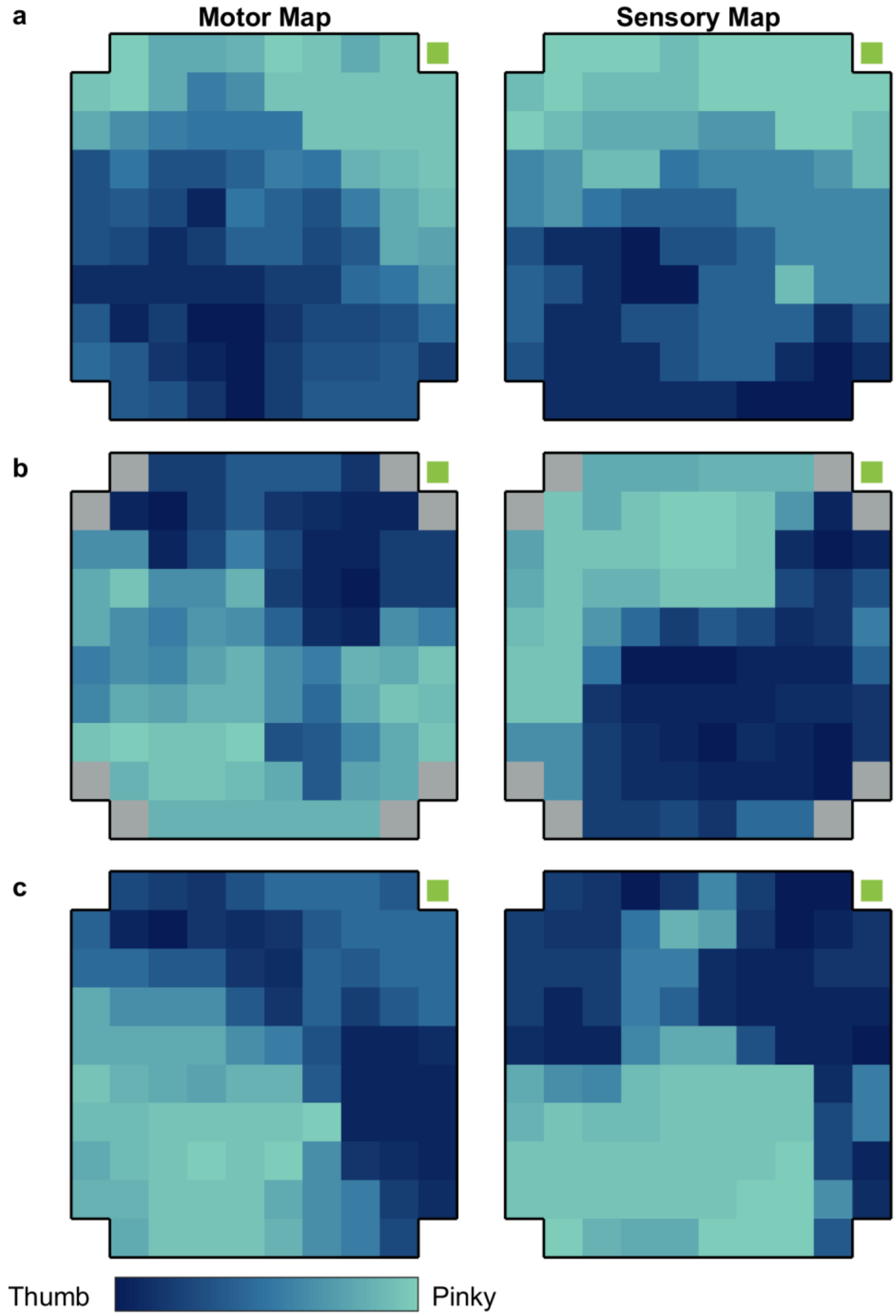


*Supplementary Figure 11. Gradients of digit preference for motor and ICMS-evoked activity. For C1 and P3, the gradient in the motor map resembles the gradient in the sensory projection map. Left: the hue denotes, for each motor channel, the Spearman correlation between the strength of the response when attempting to move each digit and the digit identity (thumb = 1, …, pinky = 5). Dark blue indicates channels that preferentially respond during attempted thumb and index finger movements, while light green indicates channels that respond preferentially to attempted movements of the ring and pinky fingers. Right: the hue denotes, for each motor channel, the Spearman correlation between the strength of the response when ICMS-activity is delivered through S1 electrodes with PFs on each digit and the digit identity (thumb = 1, …, pinky = 5). Dark blue indicates channels that preferentially respond when ICMS is delivered through S1 channels with PFs on the thumb and index finger, while light green indicates channels that respond preferentially when ICMS is delivered is S1 channels with PFs on the ring and pinky fingers. Each row shows motor and sensory projection maps for a different participant.*

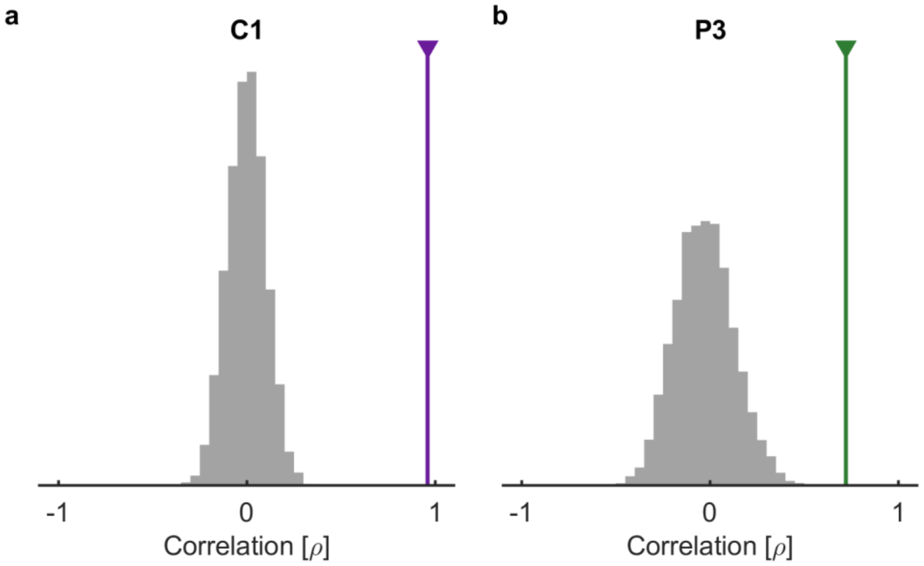


*Supplementary Figure 12. Digit preference correlation between motor task and ICMS. A| In participant C1, the strength of motor modulation to imagined movement of a digit was highly correlated with the modulation due to ICMS of sensory channels with projected fields on the same digit (median across all channels in purple) when compared to the same responses shuffled across digits and channels before calculating the correlation 10,000 times (distribution of medians in grey). B| Same as panel A but for participant P3.*

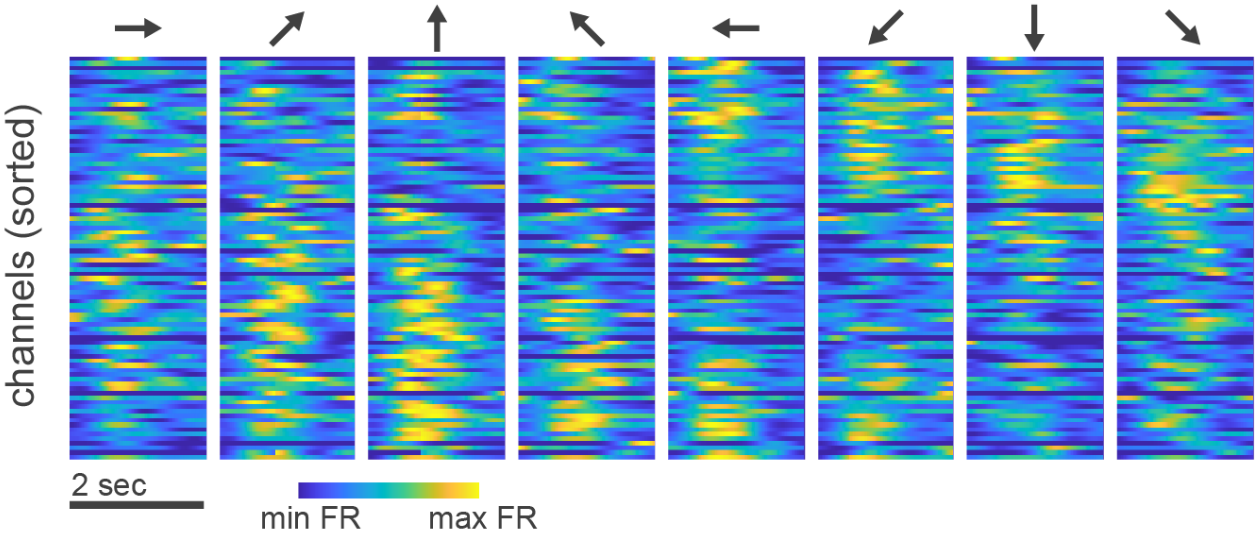


*Supplementary Figure 13. The lateral motor array in participant P2 exhibited modulated responses during center out movements. In this task, P2 rested his hand on a horizontal surface and slid it out to one of 8 peripheral targets when prompted. Channels exhibit tuning to a variety of directions across the population and enable classifying the target with 87.5% accuracy (chance = 12.5%). Thus, the lack of strong and patterned ICMS-related modulation in this participant cannot be attributed to array failure.*

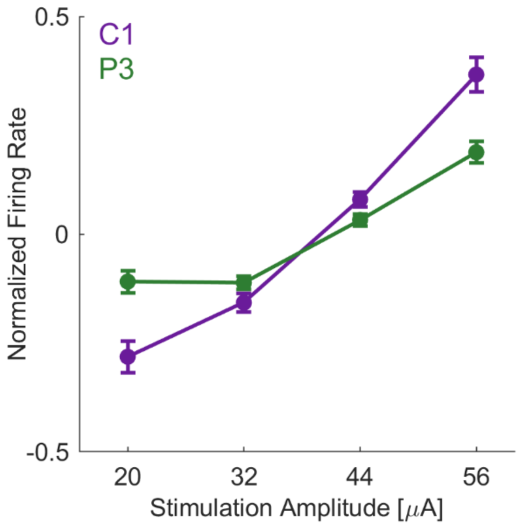


*Supplementary Figure 14. Population firing rate increases with stimulation amplitude. During the squeeze task, firing rates recorded in motor cortex generally increased with increasing amplitude of stimulation in somatosensory cortex for both participants. Plot shows mean and SEM for two participants. ($p < 0.001$ for both, Kruskal-Wallis).*

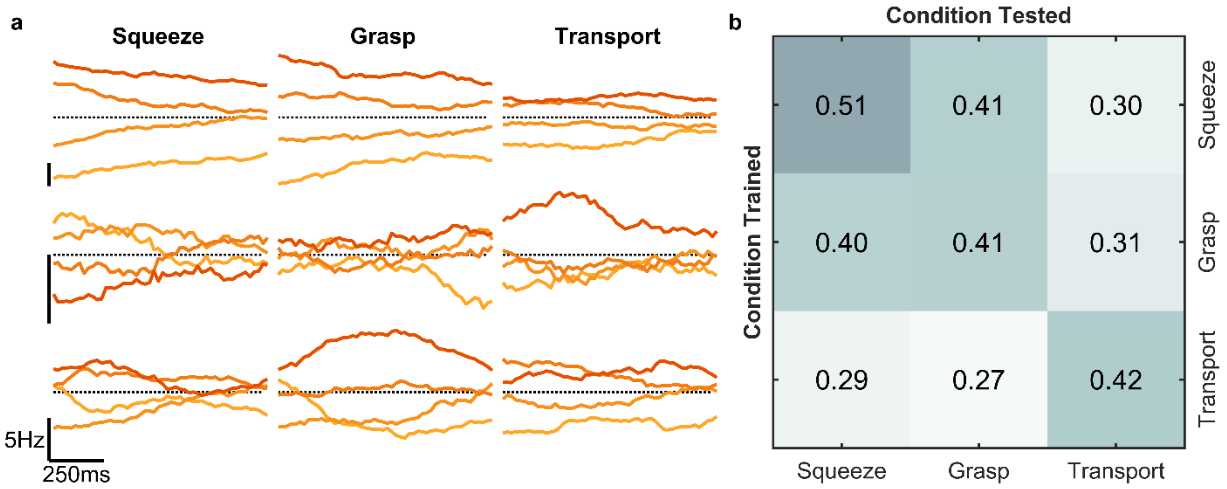


*Supplementary Figure 15. Behavioral modulation of stimulation response in P3. A| Three example motor channels exhibit different responses to four levels of ICMS across three motor conditions (squeeze, grasp, transport). B| Stimulation amplitude classifier performance. Classifiers were trained on one of the three conditions and tested on each condition (with cross-validation for within-condition classification).*

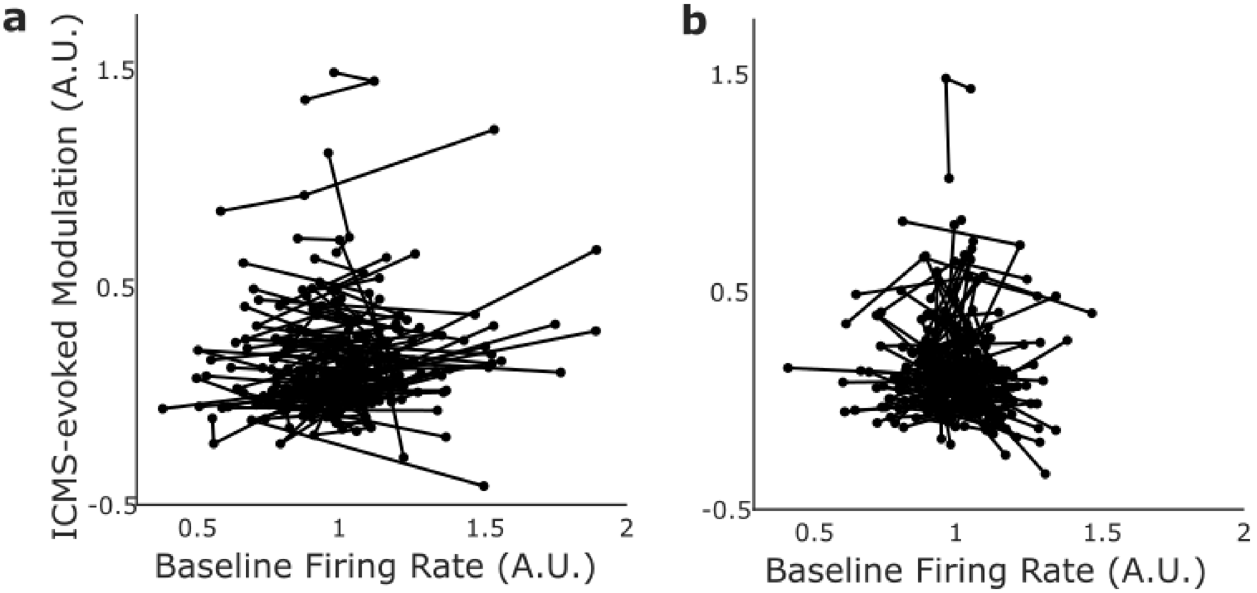


*Supplementary Figure 16. Modulation of M1 activity by ICMS amplitude vs. M1 activity evoked by the attempted movement. A| For C1 the ICMS-evoked modulation is the mean firing rate at the highest amplitude minus mean firing rate at the lowest amplitude in each task phase, divided by the average of the baseline firing rate across phases. The baseline firing rate within each task phase is divided by the baseline firing rate across task phases. Lines connect the three phases for each channel. The strength of modulation is independent of the baseline M1 activity ($R^2 = 0.012$), so the task-dependence of the ICMS-evoked activity does not reflect response saturation in M1. B| The same relationship as shown in panel A but for participant P3 ($R^2 = 0.004$).*

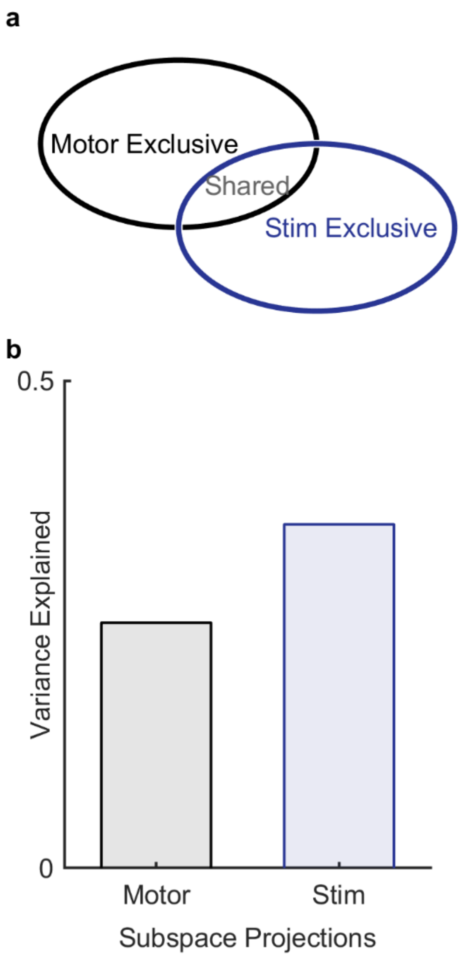


*Supplementary Figure 17. To quantify overlap between motor and stimulation subspaces, we ran two tasks. In one, we instructed the participant to attempt to grasp a virtual object at one of 4 force levels, hold it for 1 second, then release it. No stimulation was delivered during the task. In the second task, we delivered stimulation trains that were identical in duration and shape to the grasp profiles in the first task. The participant was blinded to the level of stimulation and was instructed to report the magnitude of stimulation to maintain engagement in the task. By comparing the M1 activity in the two conditions, we can extract subspaces in M1 population activity that is exclusive to the motor task (related to volitionally moving the hand) or to stimulation, as well as the subspace shared by the two tasks. A| Three subspaces were extracted: One that contains variance of the motor task, one that contains variance of the stimulation task, and one that contains the variance that is common to the two tasks (using methods from ref. [39]). B| The shared subspace captures significant variance of both motor and stimulation tasks. In other words, the activity evoked in M1 by ICMS to S1 occupies a largely overlapping subspace as does the activity evoked in M1 during attempted grasp.*
