# Supplementary material for: Microstimulation of human somatosensory cortex evokes task-dependent, spatially patterned responses in motor cortex
Source: Nat Commun. 2023 Nov 10;14:7270. doi: 10.1038/s41467-023-43140-2 (PMC10638421; doi:10.1038/s41467-023-43140-2)
Supplement: Supplementary file 3 — Reporting Summary [file 41467_2023_43140_MOESM3_ESM.pdf]

nature portfolio

Corresponding author(s): Sliman Bensmaia

Last updated by author(s): Jul 25, 2023

Reporting Summary

Nature Portfolio wishes to improve the reproducibility of the work that we publish. This form provides structure for consistency and transparency in reporting. For further information on Nature Portfolio policies, see our Editorial Policies and the Editorial Policy Checklist.

Statistics

For all statistical analyses, confirm that the following items are present in the figure legend, table legend, main text, or Methods section.

n/a	Confirmed	
☐	☒	The exact sample size (*n*) for each experimental group/condition, given as a discrete number and unit of measurement
☒	☐	A statement on whether measurements were taken from distinct samples or whether the same sample was measured repeatedly
☐	☒	The statistical test(s) used AND whether they are one- or two-sided<br>*Only common tests should be described solely by name; describe more complex techniques in the Methods section.*
☒	☐	A description of all covariates tested
☐	☒	A description of any assumptions or corrections, such as tests of normality and adjustment for multiple comparisons
☐	☒	A full description of the statistical parameters including central tendency (e.g. means) or other basic estimates (e.g. regression coefficient) AND variation (e.g. standard deviation) or associated estimates of uncertainty (e.g. confidence intervals)
☐	☒	For null hypothesis testing, the test statistic (e.g. *F*, *t*, *r*) with confidence intervals, effect sizes, degrees of freedom and *P* value noted<br>*Give P values as exact values whenever suitable.*
☒	☐	For Bayesian analysis, information on the choice of priors and Markov chain Monte Carlo settings
☒	☐	For hierarchical and complex designs, identification of the appropriate level for tests and full reporting of outcomes
☐	☒	Estimates of effect sizes (e.g. Cohen's *d*, Pearson's *r*), indicating how they were calculated

Our web collection on statistics for biologists contains articles on many of the points above.

Software and code

Policy information about availability of computer code

Data collection	Neural data was collected through the Central software suite (Balckrock Neurotech). All other data was collected through a custom software suite primarily using Python 3.0, Matlab 2019a, and C++.
Data analysis	Data was analyzed using Matlab (version 2020a or newer). Analysis code: https://github.com/CorticalBionics/ICMSconnectivity

For manuscripts utilizing custom algorithms or software that are central to the research but not yet described in published literature, software must be made available to editors and reviewers. We strongly encourage code deposition in a community repository (e.g. GitHub). See the Nature Portfolio guidelines for submitting code & software for further information.

Data

Policy information about availability of data

All manuscripts must include a data availability statement. This statement should provide the following information, where applicable:

- Accession codes, unique identifiers, or web links for publicly available datasets
- A description of any restrictions on data availability
- For clinical datasets or third party data, please ensure that the statement adheres to our policy

The deidentified data generated in this study have been deposited in the Data Archive BRAIN Initiative (DABI) under accession code https://dabi.loni.usc.edu/dsi/UH3NS107714. The data are available under restricted access for participant privacy, access can be obtained upon request to the study PIs by an investigator with a history of securely handling data resulting from human research.

March 2021

Human research participants

Policy information about studies involving human research participants and Sex and Gender in Research.

Reporting on sex and gender	All three subjects were male, which is consistent with the represented patient population (spinal cord injury, SCI) being approximately 80% male.
Population characteristics	Two participants were 28 at time of implant and the third was 57. No effects of age were expected or found. All three participants had been diagnosed with a cervical spinal cord injury (SCI), as required by the inclusion criteria.
Recruitment	Participants were recruited through voluntary research registries for people with SCI. Given the requirement of a craniotomy for inclusion in the study there is a selection bias, however we do not expect that it would influence cortical connectivity.
Ethics oversight	This study was conducted under an IDE from the FDA and with approval from the IRBs at the University of Chicago and the University of Pittsburgh.

Note that full information on the approval of the study protocol must also be provided in the manuscript.

Field-specific reporting

Please select the one below that is the best fit for your research. If you are not sure, read the appropriate sections before making your selection.

☒ Life sciences ☐ Behavioural & social sciences ☐ Ecological, evolutionary & environmental sciences

For a reference copy of the document with all sections, see nature.com/documents/nr-reporting-summary-flat.pdf

Life sciences study design

All studies must disclose on these points even when the disclosure is negative.

Sample size	Sample size of 3 subjects. Sample sizes for Brain-Computer Interface (BCI) studies in humans are limited due to the necessity of surgery and limited size of the eligible population. Most related papers only include 1 or 2 subjects.
Data exclusions	No exclusions
Replication	Experiments were replicated in 3 subjects across 2 study sites. A subset of the results did not hold for 1 of the 3 subjects, as discussed in the manuscript.
Randomization	No randomization was possible given the required surgery to implant electrodes. This study looked at anatomical connectivity, which the subjects had no voluntary control of, making randomization irrelevant.
Blinding	Blinding was not possible as there was no control group.

Reporting for specific materials, systems and methods

We require information from authors about some types of materials, experimental systems and methods used in many studies. Here, indicate whether each material, system or method listed is relevant to your study. If you are not sure if a list item applies to your research, read the appropriate section before selecting a response.

Materials & experimental systems

n/a	Involved in the study	
☒	☐	Antibodies
☒	☐	Eukaryotic cell lines
☒	☐	Palaeontology and archaeology
☒	☐	Animals and other organisms
☐	☒	Clinical data
☒	☐	Dual use research of concern

Methods

n/a	Involved in the study	
☒	☐	ChIP-seq
☒	☐	Flow cytometry
☒	☐	MRI-based neuroimaging

Clinical data

Policy information about clinical studies

All manuscripts should comply with the ICMJE guidelines for publication of clinical research and a completed CONSORT checklist must be included with all submissions.

Clinical trial registration	NCT01894802
Study protocol	Trial protocol is unavailable as it is part of an ongoing study (IDE G130082).
Data collection	Data were collected in research laboratories set up for the BCI trial at the Universities of Chicago and Pittsburgh. Data were collected over a 1-6 month period 1 year, 1 year, and 6 years after device implantation for C1, P3, and P2 respectively.
Outcomes	These results are are tangential to the primary and secondary outcomes of there overall clinical trial. Interim publication has been approved by the DSMB. The primary outcome of the ongoing trial is that the implant is safe for at least one year; all enrolled participants have exceeded this goal. The secondary outcome was functional use of the device; assessment of this outcome is still active.
